# Prevention of heterotopic ossification: an experimental study using a plasma expander in a murine model

**DOI:** 10.1186/s12893-016-0144-3

**Published:** 2016-05-04

**Authors:** Stefan M. Zimmermann, Lukas W. Schwitter, Max J. Scheyerer, Thorsten Jentzsch, Hans-Peter Simmen, Clément M. L. Werner

**Affiliations:** Department of Surgery, Division of Trauma Surgery, University Hospital Zurich, Raemistrasse 100, 8091 Zürich, Switzerland

**Keywords:** Heterotopic, Ossification, Bone, Murine, Model, Voluven, Hydroxyethyl starch

## Abstract

**Background:**

Heterotopic ossification (HO) is a frequent complication following orthopedic and trauma surgery. It often leads to substantial morbidity as many affected patients suffer from pain and joint contractures. Current prophylactic measures include nonsteroidal anti-inflammatory drugs (NSAID) and local radiation. However, several disadvantages such as delayed fracture healing and impaired ossification have been reported. For this reason, a novel approach for prevention of HO was searched for.

We hypothesized that systemic administration of hydroxyethyl starch (HES), a substance known to influence microcirculation, would reduce formation of HO in a murine model.

**Methods:**

A pre-established murine model was used where HO has been shown to develop following Achilles tendon tenotomy. Twenty CD1 mice were randomly assigned to a control (*n* = 10) or treatment group (*n* = 10). The treatment group received two intravenous HES injections perioperatively, while the control group underwent tenotomy only. After ten weeks, the mice were euthanized and micro CT scans of the hind limbs were performed. HO was manually identified and quantitatively assessed. A Wilcoxon rank sum test was used for comparison of both groups.

**Results:**

The mean heterotopic bone volume in the control group was significantly larger compared to the HES group (2.276 mm^3^ vs. 0.271 mm^3^, *p* = 0.005). A reduction of mean ectopic bone volume of 88 % was found following administration of HES.

**Conclusion:**

A substantial reduction of HO formation was found following perioperative short-term administration of HES. This work represents a preliminary study, necessitating further studies before drawing ultimate conclusions. However, this simple addition to current prophylactic measures might lead to a more effective prevention of HO in the future.

## Background

Heterotopic ossification (HO) is defined as the presence of lamellar bone in soft tissues, where bone does not normally occur [1]. It is typically found following fractures and dislocations, burns, as well as operative procedures. Apart from some genetic origins, it is most commonly observed in the setting of traumatic brain injury [2]. Its predominant site is within the soft tissue surrounding joints, mostly affecting the hip. Much is yet unknown concerning the pathophysiology leading to HO, but several contributing factors have been identified. It is believed that inappropriate differentiation of pluripotent mesenchymal stem cells into osteoblastic stem cells plays an important role, which among others is triggered by local tissue hypoxia [3]. In case of stimulation, these stem cells begin to differentiate into osteoblasts with consequent osteoid formation [1]. In this context, previous studies have demonstrated a clear correlation between a hypoxic microenvironment and HO development [4]. Furthermore, an inducing agent and a permissive environment seems to be necessary as described previously by Balboni and colleagues [2]. Urist et al. postulated a small hydrophobic bone morphogenetic protein as a further causative agent [5]. It was suggested that this protein is liberated from normal bone in response to venous stasis, inflammation, or in case of a disease of the connective tissue attachments to bone [6]. All these conditions are often found in immobilized patients as well as following trauma. Finally, Prostaglandin E2 has been shown to influence the differentiation of stem cells as well [7, 8].

As of today, surgical removal is the only treatment option once HO has occurred. However, postoperative results are often dissatisfying as high recurrence rates after excision have been reported and frequently complicate the further course of treatment. Therefore, an effective prophylactic regimen is of great interest. Current prophylactic measures generally adhere to one or more of the following three principles: disrupting the relevant inductive signaling pathways, altering the relevant osteoprogenitor cells in the target tissue, or modifying the environment conducive to heterotopic osteogenesis. The latter can be influenced by optimizing microcirculation, which prevents local tissue hypoxia. In this context, hydroxyethyl starch (HES, Voluven®) has been shown to reduce local tissue hypoxia by enhancing tissue oxygen tension and regulating microcirculation in a murine model [9, 10].

Hoffmann et al. studied the effects of volume support during microcirculatory disorders in an animal model. They examined leukocyte-endothelial cell interaction (LE), functional capillary density (FCD) and macromolecular leakage as indicators of microcirculation using intravital microscopy. A significantly increased FCD and less macromolecular leakage was found following the administration of HES compared to a saline and control group indicating a positive effect on microcirculation [9]. In an ischemia/reperfusion model in rabbits, one group was infused with 0.9 % saline and the other group with HES. Later, muscle biopsies were performed and significantly lower myeloperoxidase (MPO) levels were found in the HES group, demonstrating a positive effect on oxidative stress compared to the saline infusion group [10].

In light of this evidence, the aim of our current study was to quantitatively assess HO formation following intravenous administration of HES. We hypothesized that intravenous administration of HES would lead to a decrease in HO formation.

## Methods

### Animal model

Prior to the investigation, approval from the relevant Swiss authorities was acquired (Kantonale Tierversuchskommission Zürich, Switzerland, approval number 175/2008), and experimental animal investigation guidelines of the European Union (Directive 2010/63/EU) were strictly adhered to. A pre-existing well-established murine model was chosen, where HO reliably occurs following Achilles tendon tenotomy [11–13]. The site of subsequent HO formation is within the soft tissue surrounding the tenotomy.

**Fig. 1:**
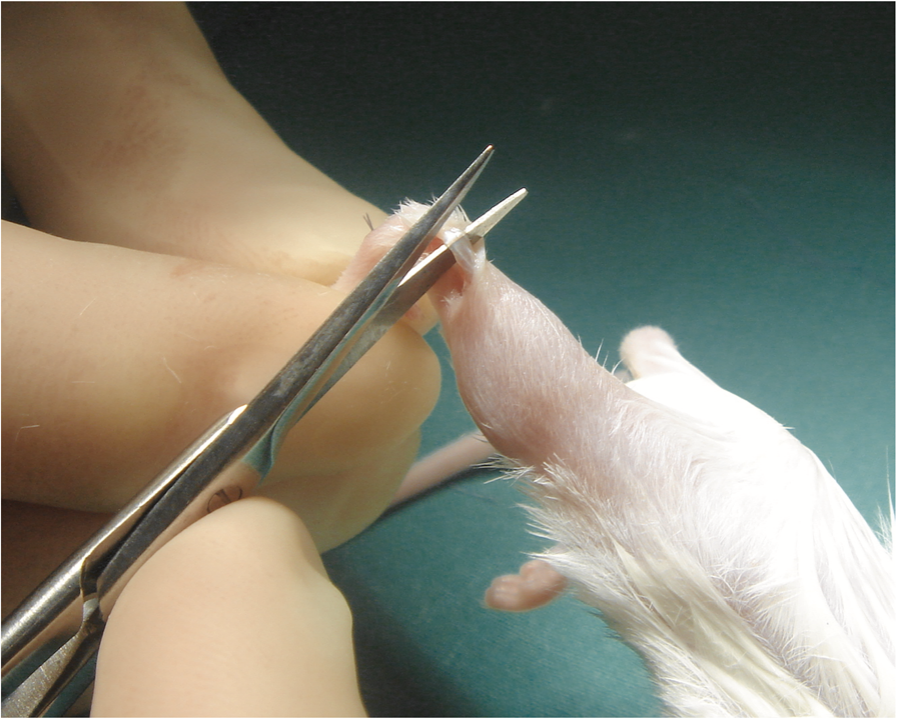
Mouse hind limb Achilles tendon tenotomy

Although the model described does not require a specific strain or breed of mice, only male specimens were used. This ensured comparability to other similar investigations, where only male subjects were used as well. Cluster of differentiation 1 (CD1) mice were chosen as they are not genetically modified, bred locally, and finally handling was facilitated as they are rather large animals. Identification was carried out by individualized markings on the tail. All specimens underwent bilateral midpoint Achilles tendon tenotomy through a posterior approach (Fig. 1). The skin was subsequently closed using non-absorbable sutures. Anaesthesia consisted of Isoflurane (Baxter International Inc., USA), 5–2 % in oxygen at a flow rate of 400 ml/min via a nose cone, combined with subcutaneous administration of Buprenorphine (Temgesic, Reckitt Benckiser, Slough, Great Britain).

Following this procedure, the animals were randomly assigned to one of two groups: A control group (*n* = 10) and a treatment group (*n* = 10). This sample size was based on convenience. The control group underwent Achilles tenotomy only. The treatment group additionally received 200 μl HES (Voluven, Fresenius Kabi, Bad Homburg, Germany) intravenously by means of a tail vein injection immediately postoperatively as well as on the first postoperative day. Intravenous substance application in a murine model is best carried out either via the internal jugular vein by micro-surgically inserting a catheter, or by means of administration via the tail vein. The latter is easier to perform, but drawbacks include limited applications, as tail vein thrombosis commonly occurs after multiple injections. We therefore chose to administer HES by means of a total of two consecutive injections. The dose was chosen comparable to a standard administration in humans (7 ml/kg). This was followed by 10 weeks of cage activity only for both groups.

Acetaminophen syrup was used for postoperative analgesia (Dafalgan 3 %, 200 mg/kg, Bristol-Myers Squibb SA, New York City, NY, USA), for 1–3 days [14].

All animals were evaluated several times a day postoperatively for possible signs of distress, pain and discomfort such as apathy, shivering as well as reduced chow and water intake. The findings were recorded on a score-sheet. If any such signs were noted, treatment with acetaminophen was extended and early abortion would have been considered if any of these signs had persisted. At ten weeks post-surgery, all mice were euthanized and the limbs harvested.

### Assessment

**Fig. 2:**
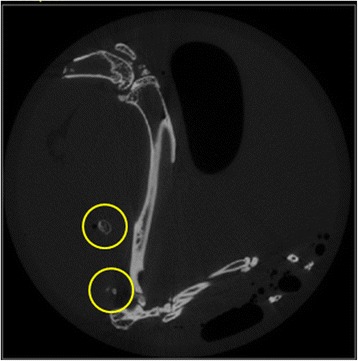
Micro CT 2D image of mouse hind limb with HO

**Fig. 3:**
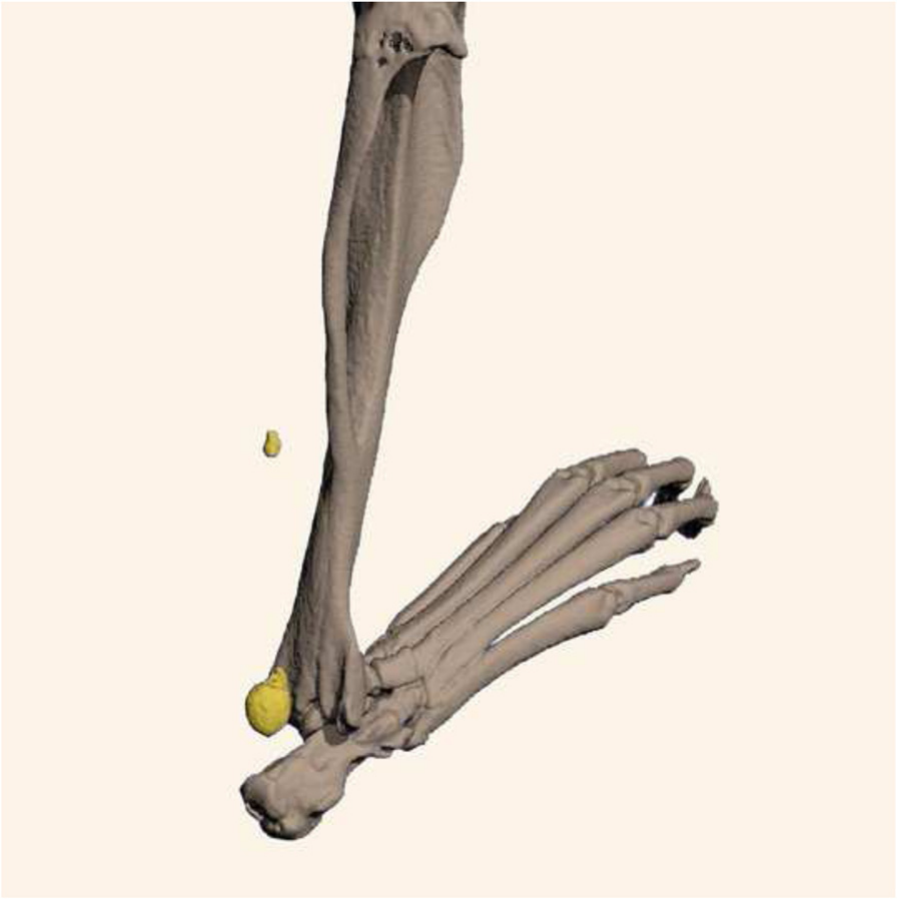
Micro CT 3D reconstruction of mouse hind limb with HO

A micro-computed tomography (CT) scan (SCANCO Medical Micro CT, Zurich, Switzerland) of all specimens was performed with a resolution of 30 μm. The generated images showed a series of 2-dimensional slices through the specimen (Fig. 2). Using a fixed threshold procedure, skeletal and ectopic bone were segregated from the background. Heterotopic bone was then manually identified and marked. Per definition, HO was identified as any bone in the soft tissue with a density that was at least equal to that of spongy skeletal bone. Afterward, each limb was three-dimensionally reconstructed and the volume of HO was visualised (Fig. 3). Thereafter, the volume of heterotopic bone was calculated using the quantitative bone analysis software provided with the micro-CT system.

### Statistical analysis

Statistics were performed in cooperation with the division of biostatistics at the Institute for Social and Preventive Medicine of the University of Zurich, Switzerland. Data were given as heterotopic bone volume in mm^3^. The analysis was performed with SPSS (Version 2.0, IBM, Chicago, IL, USA). Bone volume was examined using descriptive statistics (ANOVA) and as data was not normally distributed, differences between the groups were identified using the Wilcoxon rank sum test. Categorical data was assessed with the Fisher’s exact test. The level of significance was set at *p* < 0.05.

## Results

The first two specimens died perioperatively which was attributed to anaesthesiologic reasons. All subsequent procedures could be carried out without complications. All of the remaining 18 animals survived and were randomly allocated to either the control or treatment group. No further severe adverse events were recorded. Acetaminophen use and postoperative ambulation were comparable in both groups. All limbs were harvested at ten weeks postoperatively and scanned to assess HO formation.

**Fig. 4:**
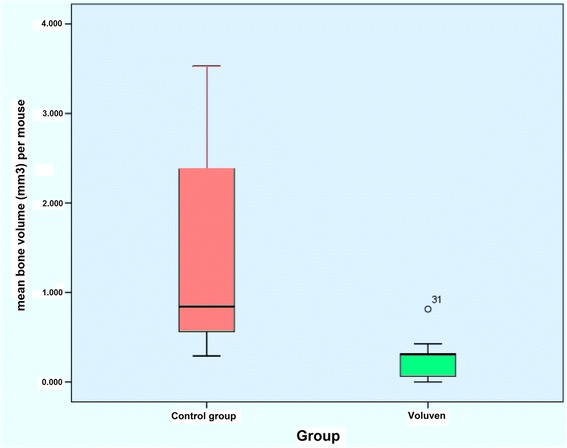
Box-Whisker plot showing HO volume for both groups. The natural logarithm of data is presented due to the non-normal distribution

**Fig. 5:**
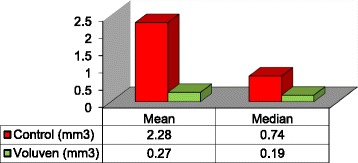
Mean and median bone volume (mm3) for control and treatment group

Overall, HO occurred in 32/36 (88.9 %) limbs, while no HO was observed in 4/36 (11.1 %) limbs. It occurred in 17 of 18 (94.4 %) specimens in the control group and in 15 of 18 (83.3 %) specimens in the treatment group. This difference was, however, not significant (*p* = 0.603). In both groups alike, HO formation occurred in several different independent small areas and did not typically consist of one single continuous mass. There was no statistical difference between both groups regarding the number of islets of HO that formed. There was a highly significant difference in the cumulative volume of HO between both groups with decreased HO volume in the treatment group compared to the control group (*p* = 0.005, Fig. 4). In the treatment group, the mean cumulative HO volume was 0.271 mm^3^ (range, 0–8.27 mm3, SD 0.269 mm^3^, Fig. 5) compared to 2.276 mm^3^ in the control group (range, 0–17.0 mm^3^, standard deviation (SD) 4.047 mm^3^). Following administration of HES, a subsequent substantial reduction of mean HO bone volume of 88 % was therefore recorded.

## Discussion

Prevention of heterotopic ossification is of great clinical interest as it is a complication commonly seen following trauma, orthopaedic surgery and particularly joint arthroplasty [15]. It may lead to pain and joint contractures. Prevention and treatment of HO is based on three principles: (1) disrupting the relevant inductive signaling pathways, (2) altering the relevant osteoprogenitor cells in the target tissue and (3) modifying the environment conducive for heterotopic osteogenesis [2, 3]. NSAIDs and radiation therapy are currently considered the gold standard in HO prevention [16, 17]. They act by modifying the microenvironment as they reduce the associated inflammatory process involved in HO formation. Despite their efficacy, complete prevention of HO often cannot be ensured. Furthermore, use of NSAIDs is controversial due to potentially deleterious gastrointestinal side effects, impaired fracture healing and possibly decreased implant ingrowth [18–20]. These are two particularly unfavorable effects in a trauma setting or following joint arthroplasty.

To date, none of these undesirable effects have been documented following administration of HES. Furthermore, HES is already in use today for volume resuscitation in a trauma setting to reduce the need for allogenic blood transfusion and improve rheology by decreasing the blood viscosity [21, 22]. Previous studies demonstrated reduced local tissue hypoxia following the administration of HES by enhancement of tissue oxygen tension and regulation of microcirculation in animal models [9, 10].

A hypoxic microenvironment appears to be an important contributing factor in the formation of ectopic bone. In this context, Olmsted et al. have studied the microenvironment surrounding the site where HO develops and reported that brown adipocytes started accumulating in this area through generation of hypoxic stress within the target tissue, a necessity for the differentiation of stem cells into chondrocytes, subsequently leading to heterotopic bone formation [23].

A key factor in differentiation of mesenchymal stem cells to chondrocyte cells is HIF-1 alpha [24]. It directly influences chondrocyte-specific gene expression and subsequent differentiation of mesenchymal stem cells to chondrocytes [25].

With pre-existing evidence indicating a beneficial effect of HES on local soft tissue microenvironment and microcirculation, we set out to investigate the clinical effect of HES on HO formation in a murine model. The model used has been shown to consistently produce islets of ectopic bone in the surrounding soft tissue following a midpoint Achilles tendon tenotomy [11]. Mouse hind limbs were harvested ten weeks postoperatively and formation of HO was assessed with use of Micro CT. Contrary to the original publication of McClure and colleagues, where HO was reported in 100 % of the tenotomized specimens, we found an overall occurrence of ectopic bone following this procedure in 88.9 % of all limbs (*n* = 32/36), while no HO was observed in 11.1 % (*n* = 4/36). It occurred in all but one of the specimens in the control group (94.4 %, *n* = 17/18), while three specimens in the treatment group (16.7 %, *n* = 3/18) showed no signs of HO bone formation at all. The complete absence of HO in three cases in the treatment group was desired, but due to the small sample size, this difference may have not been statistically significant (*p* = 0.603). A highly significant difference in mean ectopic bone volume could be found however, with a mean HO bone volume of 2.276 mm^3^ in the control group compared to 0.271 mm^3^ in the treatment group. A remarkable reduction of mean volume of ectopic bone formation of 88 % was therefore found following the delivery of HES.

Beside other previously investigated experimental substances influencing the formation of ectopic bone formation, such as Echinomycin or Imatinib, the great advantage of HES is the substantially better side effect profile with a low incidence of undesirable effects [12, 13, 26]. HES is already implemented in daily use for volume resuscitation in a trauma or surgical setting involving hemorrhage or shock.

Although the volume of HO formation could be reduced, it was not possible to completely prevent ectopic bone formation in most cases (83.3 % *n* = 15/18). We assume that multiple additional involved signaling pathways, which were not influenced by HES, may account in part for this. Therefore, application of just one substance seems unlikely to completely prevent ectopic bone formation.

Further studies are necessary to evaluate a possible combination of administered substances for a more effective prevention of HO, possibly by influencing other contributing factors such as increased release of Prostaglandin E2, hypercalcemia, changes in symptomatic nerve activity and the disequilibrium of parathyroid hormone and calcitonin [3, 7, 27].

To our knowledge, this is the first study to describe the clinical effect of HES on HO formation in a standardized model. However, this investigation has its limitations.

As our study utilized a murine model, the applicability of our results in a clinical setting has yet to be investigated. Further, the small sample size of ten mice per group and nine mice per group at final follow-up may be criticized. As this work can be regarded as a pilot study, the sample size was chosen as small as possible. However, we believe that this does not substantially influence our results as we were able to demonstrate a statistically significant difference in HO formation between the groups despite the small sample size. Although previous investigations have brought up substantial evidence in order to explain the above mentioned involved signaling pathways, which are involved in HO formation, the exact mechanism by which HES led to a reduction of HO formation in our current study remains speculative and has yet to be explored. The present study merely evaluated the clinical effect of HES. Furthermore, a third study arm would be desirable with administration of intravenous saline as a control as well as a comparison with current standard treatment with a non-steroidal anti-inflammatory drug, such as Indomethacin, in order to better understand the potential of HES to reduce HO formation.

Finally, it is known that current preventive measures for HO may interfere with physiological fracture healing as well as implant ingrowth. The effects of HES on fracture healing as well as implant ingrowth have yet to be investigated as well.

Beside these limitations, our results are the first to demonstrate a clinical effect of intravenous perioperative administration of HES on HO formation with a relevant decrease of ectopic bone. Finally, we are aware that this novel approach is merely the groundwork in an area warranting further research to assess the potential of HES in patients. These promising preliminary results, however, may lead to a more effective prevention of HO in the future by a simple addition to current prophylactic measures.

## Conclusions

Formation of heterotopic bone is a frequent complication following trauma, burns and orthopedic surgery. Treatment is often challenging, as high recurrence rates following surgical excision have been reported. Current prophylactic measures have proven efficacy, but may not always completely prevent HO from occurring. For this reason, we searched for a novel approach to further improve prevention of HO.

We found a substantial decrease in heterotopic bone formation following perioperative short-term administration of hydroxyethyl starch in a standardized murine model. This work represents a preliminary study as the mechanism of action of HES, as well as applicability in humans, have yet to be investigated. However, this simple addition to current prophylactic measures could lead to a more effective prevention of HO in the future.

## Ethics approval

Local ethics approval was obtained (Kantonale Tierversuchskommission Zürich Number 175/2008).

Experimental animal investigations guidelines of the European Union (Directive 2010/63/Eu) were strictly adhered to.

## Availability of data and materials

All data of the present investigation can be obtained from the corresponding author.

## Abbreviations

CD1: cluster of differentiation 1
FCD: functional capillary density
HES: hydroxyethyl starch
HO: heterotopic ossification
LE: leukocyte-endothelial cell interaction
MPO: myeloperoxidase
NSAID: non steroidal anti-inflammatory drug
